# The Effect of ACE Inhibitor on the Quality of Life amongst Patients with Cancer Cachexia

**DOI:** 10.31557/APJCP.2020.21.2.325

**Published:** 2020

**Authors:** Mehdi Dehghani, Mehdi Mirzayi, Pouya Farhadi, Alireza Rezvani

**Affiliations:** 1 *Hematology Research Center, Department of Hematology and Medical Oncology, *; 2 *Department of Internal Medicine, *; 3 *Cardiovascular Research Center, Shiraz University of Medical Sciences, Shiraz, Iran. *

**Keywords:** ACE inhibitor, cancer cachexia, gastric cancer, Captopril

## Abstract

**Background:**

ACEI (Angiotensin Converting Enzyme Inhibitors) inhibits tumor growth and development. Different mechanisms have been proposed for this matter, including the inhibition of enzymes that are involved in extracellular matrix degradation, matrix metalloproteinase (MMP) and etc. The present study was designed with the aim to investigate the effects of low dose ACEI on the Quality of Life (QoL) of non-hospitalized gastric cancer patients with cachexia.

**Materials and Methods:**

This study was a single-blinded randomized controlled clinical trial conducted in clinics affiliated with Shiraz University of Medical Sciences (SUMS). All participants were patients with gastric cancer in cancer cachexia step aged 40-80 years old who had referred to our clinics from October 2013 to April 2014. In the intervention group, patients were assigned to receive ACEI (Captopril) and the placebo group served as control and received placebo during the same time course. They were asked questions in order to fill out QLQ-C30 (Persian Version) questionnaire 3 times; baseline, 1 and 2 months after their first visit.

**Results:**

The mean age of patients was 60.55 ± 12.07 (range 31-80) years and the mean BMI of the patients was 17.21 ± 2.31. In the ACEI group, physical functioning and fatigue score changes were significant 1 and 2 months after treatment. The mean of fatigue score decreased significantly in the placebo group. Overall, global health status scores significantly increased in both groups, but other items of QoL did not change significantly.

**Conclusion:**

Overall, our results showed that Captopril does not have a significant positive effect on QoL of patients with cancer cachexia.

## Introduction

Loss of weight and appetite, reduction in physical function and intolerance to anti-cancer therapy is an inevitable condition amongst cancer patients. Cachexia is a weakening condition which involves involuntary weight loss. Cancer patients with cachexia have poor prognosis due to higher chance of infection and inflammatory diseases. Cachexia comes from the Greek words of ‘kako’ and ‘hexis’ meaning ‘bad’ and ‘condition’. Cachexia presents itself with anorexia, involuntary weight loss, poor performance that ultimately leads to death (Suzuki et al., 2013).

Cachexia can have a severe impact on the patient’s confidence and quality of life (QoL) (Inui, 1999). The pathogenesis of cachexia is unclear, but it was stated that pro-inflammatory cytokines might be released from tumors’ cells due to inflammation, which might be secreted from the tumor itself (Fearon et al., 2011).

There is no definite treatment for cachexia and physicians disagree on its management, and only progestines and corticosteroids are advised. However, they cannot prevent muscle loss and due to their side effects, long-term usage is not advised (Fearon et al., 2013).

In animal model, angiotensin II (AngII) caused anorexia and cachexia, which led to lose of 18-26% of body weight in rats, during a short period of time in comparison with controls (Brink et al., 1996). Brink et al., (2001) stated that angiotensin I (AngI) stimulates protein deterioration in muscles by converting into Ang II, which was due to an inhibitory effect on the IGF-I system. AngII might increase protein deterioration with inhibitory effect on the autocrine IGF-I system. 

Tumor growth and development is inhibited by Angiotensin-Converting Enzyme Inhibitors (ACEIs), which occurs in different ways; including inhibition of enzymes with a role in extracellular matrix deterioration, matrix metalloproteinase (MMP) and etc. Matrix deterioration is entirely under the control of MMPs which is required for differentiation, angiogenesis, adhesion and metastasis with molecular bioactive processing (Lindberg et al., 2003) 

Tumor responses and overall survival are the traditional outcomes, used to assess the effectiveness of therapy in cancer patients. Within the past two decades Health Related Quality of Life (HRQoL) has become an important factor for evaluating new modalities in cancer therapy. HRQoL gives patients suitable knowledge and awareness with respect to benefits of therapy; hence, the accuracy of measurements and having a standard questionnaire when collecting data is vital (Pallis and Mouzas, 2004).

Considering that there are few studies that have explored the effects of Ang II blockers for improving the QoL in cachectic cancer patients, the present study was designed to investigate the effect of ACEI on the QoL of non-hospitalized patients with cancer cachexia.

## Methods and Materials

*Trial design*

The present study is a randomized single-blinded, placebo-controlled trial, conducted in clinics affiliated with Shiraz University of Medical Sciences (SUMS). The study protocol was approved by the institutional review board (IRB) of SUMS, and was approved by the local Ethics Committee of SUMS. After explaining the study objectives, all the participants gave their written informed consent. The trial was registered in the Iranian Clinical Trials Registry (IRCT2014050511375N3; www.irct.ir).

*Participants*

All participants were gastric cancer patients with cancer cachexia aged 40-80 years old, who had referred to clinics affiliated with SUMS from October 2013 to April 2014. Cachectic patients are defined as BMI (Body Mass Index) ≤18 kg/m^2^ and those with muscle wasting or only temporal wasting. The exclusion criteria are as follows: patients unable to continue the treatment, patients with BMI ≥18 kg/m^2^ without temporal or muscle wasting, those with GFR<50 ml/min, positive history of other comorbidities (RF, CHF, COPD, CAD), and patients with Hb<9.

*Intervention and outcomes*

Participants in the case group were assigned to receive ACEI (Captopril) starting with 6.25 mg, three times a day and increasing the dosage, if could tolerated to 12.5 mg two/three times a day (ACEI group). The control group was given starch pills, manufactured by Shiraz Pharmacology School as the placebo and the treatment course was similar to the ACEI group.

Patients with cancer Cachexia were asked to fill out QLQ-C30 (Persian Version) questionnaire 3 times, once at the baseline, 1 and 2 months after treatment, in order to obtain the values of ACEI on the QoL (Montazeri et al., 1999) 

The QLQ-C30 questionnaire consists of 30 questions in 3 categories of global health status/quality of life, functional scales, and symptom scales. In addition, demographic data were separately collected. Each category has different items with different interpretations and each item is evaluated by asking specific questions. As an example, dyspnea is categorized as a symptom scale, which is evaluated by asking the patient whether he/she had a shortness of breath. 

*Randomization*

The patients were randomly allocated into two study groups, using a computer-based random digit generator, based on the registration number of the patients (the orders are referred).

*Statistical Analysis*

In order to achieve 90% power to detect significant differences in QoL improvement, 18 patients were required in each study group (p<0.05, two-sided). To compensate for possible patients dropout, we recruited 50 participants in total. All statistical analyses were performed, using the statistical package for social sciences version 17.0 (SPSS Inc., Chicago, IL, USA). The baseline characteristics of both groups were compared using X^2^ tests or Fisher’s exact test for proportions and the unpaired t-tests for means. We also used ANOVA to compare QoL of the case and placebo groups before and after treatment. Data are reported as means ± SD. A two-sided p-value less than 0.05 was considered to be statistically significant. 

**Table 1:** Baseline Characteristics of Participants

Variables	ACEI Group (20)	Placebo Group (20)	P value
Demographics			
Age (years) *	56.4±12	64.7±10	0.08
Gender			0.73
Male†	14 (70%)	13 (65%)	
Female†	6 (30%)	7 (35%)	
Weight (Kg)	51.7±8.6	57.2±1.8	0.37
BMI (Kg/m^2^) *	17.6±3.1	17.1±1.9	0.71
Chemotherapy Regimens			0.51
OX†	13 (65%)	10 (50%)	
EOX†	4 (20%)	6 (30%)	
EOF†	1 (5%)	3 (15%)	
DCF†	1 (5%)	1 (5%)	
Folfox†	1 (5%)	-	
Medications			
Celecoxib†	2 (10%)	3 (15%)	0.86
Zinc†	12 (60%)	11 (55%)	0.79
Multivitamin†	4 (20%)	6 (30%)	0.43

*, Average; Standard Deviation; †, Frequency and Percentages; OX, Oxaliplatin; EOX, Epirubicin + Oxaliplatin + Xeloda; EOF, Epirubicin + Oxaliplatin + 5-FU; DCF, Docetaxel + Cisplatin + 5-FU; Folfox, FOLolinic acid + 5-FU + Oxaliplatin

**Table 2:** Comparing the Mean Scores of QLQ-C30 between Two Groups in Baseline

Variables	ACEI Group (20)	Placebo Group (20)	P value
Functional scales			
Physical functioning	61±27.1	66.6±20.9	0.46
Role functioning	85±18.6	75.3±17.4	0.09
Emotional functioning	67.5±24.4	81.6±22.2	0.06
Cognitive functioning	81.6±17.1	74±15.1	0.14
Social functioning	73.3±25.5	62.5±46.1	0.36
Symptom scales			
Fatigue	46.6±27.1	55.5±25.2	0.29
Nausea and vomiting	21.6±30.1	35.8±35.5	0.18
Pain	41.6±34.4	41.6±32.2	0.9
Dyspnea	13.3±16.7	16.3±22.9	0.6
Insomnia	36.6±34.1	41.6±37.2	0.66
Appetite loss	50±35.1	50±31.5	0.9
Constipation	28.3±39.4	30±26.2	0.87
Diarrhea	13.3±22.6	11.6±19.5	0.81
Financial difficulties	46.6±39.5	40±33.5	0.56
Global health status	56.2±21.1	44.1±28.6	0.13

**Table 3:** Comparing the Mean Scores of QLQ-C30 between Each Group; Baseline, 1 and 2 Months after first Visit

Variables	ACEI Group			P	Placebo Group			P
	Baseline	1 Month	2 Months		Baseline	1 Month	2 Months	
Functional scales								
Physical functioning	61±27.1	63.6±47.2	75±26.8	0.03	66.6±20.9	63.3±25.4	67.3±27.7	0.13
Role functioning	85±18.6	74.1±33.9	63.3±24.1	0.56	75.3±17.4	60.8±27.2	65.8±24.3	0.51
Emotional functioning	67.5±24.4	60.8±28.6	77.1±20.1	0.31	81.6±22.2	67.1±28.6	74.1±23.5	0.13
Cognitive functioning	81.6±17.1	74.1±27.8	80.8±19.7	0.78	74±15.1	76.6±23.8	78.3±21.6	0.12
Social functioning	73.3±25.5	56.6±34.3	75.8±21.9	0.12	62.5±46.1	73.3±19.7	75.8±20.5	0.89
Symptom scales								
Fatigue	46.6±27.1	40.1±32.9	32.2±27.6	0.001	55.5±25.2	45±27.8	41.8±26.8	0.03
Nausea and vomiting	21.6±30.1	28.3±31.1	22.5±31.6	0.39	35.8±35.5	25.8±28.3	24.1±31.7	0.19
Pain	41.6±34.4	44.1±35.5	29.1±30.5	0.19	41.6±32.2	38.3±27.6	30.8±28.2	0.12
Dyspnea	13.3±16.7	15±31.4	8.3±23.8	0.28	16.3±22.9	16.6±20.2	13.3±25.1	0.67
Insomnia	36.6±34.1	40±38.3	35±33.2	0.6	41.6±37.2	35±31.4	33.3±26.4	0.52
Appetite loss	50±35.1	45±32.9	35±33.2	0.11	50±31.5	43.3±34.3	33.3±26.4	0.06
Constipation	28.3±39.4	23.3±26.7	15±20.1	0.11	30±26.2	23.3±24.4	20±27.3	0.07
Diarrhea	13.3±22.6	21.6±32.9	15±31.4	0.71	11.6±19.5	20±27.3	11.6±22.3	0.43
Financial difficulties	46.6±39.5	55±39.4	35±31.4	0.23	40±33.5	43.3±34.3	35±29.5	0.26
Global health status	56.2±21.1	52.9±29.8	66.2±23.3	0.02	44.1±28.6	50.4±27.8	62.9±17.9	0.03

**Figure 1:**
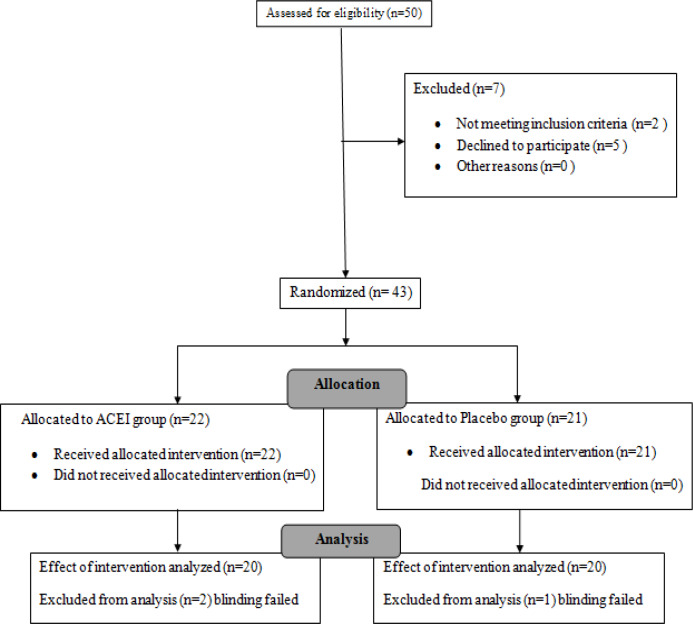
CONSORT 2010 Flow Diagram for this Randomized, Single-Blinded, Placebo Controlled Clinical Trial of Effectiveness of Angiotensin Converting Enzyme Inhibitor on the Quality of Life of Non-Hospitalized Patients with Cancer Cachexia

## Results

Out of the 50 patients assessed for eligibility, two did not meet the inclusion criteria and five declined further participation. Thus, the final number of patients being randomized into two study groups was 43. Blinding failed for three patients allocated to receive treatment, and finally 20 patients were enrolled in each group (Figure 1).

The mean age of patients was 60.55 ± 12.07 (range 41-80) years, and the mean BMI of the patients was 17.21 ± 2.31 kg/m^2^. Amongst the patients, there was 27 (67.5%) male and 13 (32.5%) female. The baseline characteristics of the patients are reported in three main ategories of demographics, chemotherapy regimens and medications (Table 1).

There was no significant difference between the two study groups regarding baseline characteristics. Moreover, we compared QoL amongst patients based on QLQ-C30 questionnaire at the baseline. There was no significant difference between ACEI and the placebo group, regarding items of QLQ-C30 at the baseline (Table 2). 

In ACEI group, physical function score had increased significantly 1 and 2 months after treatment course (P=0.03). Patients in the placebo group also experienced significant reduction in fatigue scores after treatment course (P=0.03); however, patients in ACEI group had significantly lower scores for fatigue (P=0.001). In addition, global health status scores increased significantly in both groups. Meanwhile, global health status changes in ACEI group were more than that of the placebo group. There were no statistically significant differences between the baseline, 1 and 2 months after treatment, regarding other items of QLQ-C30 in each group. Table 3 shows the changes in QoL in each group.

The mean weight of participants after 2 months reached 52.58±7.42 kg in ACEI group in comparison to baseline (51.7±8.6)which was not significant (P=0.27). Meanwhile the mean weight of participants in the control group 55.21±7.71 kg in placebo group after 2 months and decreased significantly (P=0.003).

## Discussion

Cancer cachexia is multifactorial syndrome, which single treatment modality cannot be effective, and due to this interdependency, there should be multidisciplinary treatment approach to increase patient’s QoL (Suzuki et al., 2013).

The best way to treat cancer cachexia is to cure the underlying causes, but in adults with advanced malignancy, this is a farfetched accomplishment. In the management of cancer cachexia, appetite, body weight and survival are the ultimate endpoints; hence, the goal of treatment should be to prevent body weight loss and muscle mass, using different pharmacological agents (Inui, 2002).

Nutritional support without the aid of pharmacological agents is unlikely to affect the overall survival in advanced cancer. But then again, combining treatment that intends to control tumor inflammation as well as metabolic abnormalities by concentrating on energy wasting might have several advantages. Therapeutic strategies that target the mediators of the catabolic responses, such as cytokines and eicosanoids or metabolic regulation might offer more assurance in the future; in addition, early detection and intervention might be more helpful (Bosaeus, 2008).

According to studies, inflammation is the main cause of cachexia. Hence, producing prostaglandins that can cause pain and inflammation, inhibited by Aspirin or NSAIDS such as ibuprofen that are non-selective blockers of cyclooxygenase pathway (COX-2 pathway) might be effective in controlling cancer inflammation and also cachexia (Madeddu et al., 2012). These drugs also inhibit genesis of thromboxan A2 (TXA2) in platelets by preventing platelet aggregation. Several authors claimed that NSAIDS treatment in cancer cachexia is sufficient, as a routine treatment (Rogers et al., 2011).

A number of trials stated that carbohydrate intake and metabolic efficiency during exercise stimulated by low dose insulin treatment (0.11±0.05 units/kg/day), resulted in increased survival in cancer cachexia patients. On the other hand, fat free mass, increased exercise and spontaneous physical activity were not changed. The act of basic metabolic reversing in cancer cachexia is not clear, yet.

In theory, Angiotensin II will increase in cancer patients and the raise in protein degradation and ubiquitin ligases induced by Ang II in-vivo were shown to be blocked by muscle specific expression of IGF-I. It seems that the downregulation of IGF-I has relation with muscle wasting (Sanders et al., 2005).

There are limited studies regarding ACE inhibitors on QoL amongst cancer patients, but there is a phase two study on the ability of ACE inhibitor (captopril)to alter the incidence of pulmonary damage after radiation therapy and also quality of life in patients with lung cancer.(Small, James et al., 2018) 

In cachectic patients with Congestive Heart Failure (CHF), combination therapy of the angiotensin-converting enzyme (ACE) inhibitor “enalapril”, Digoxin and diuretic increase subcutaneous fat and muscle bulk together with a significant raise in plasma albumin (Adigun and Ajayi, 2001). Moreover, it was shown that ACEI treatment can slow or stop reduction in muscle strength in elderly women with hypertension without CHF (Onder et al., 2002). Also, ACEI degrades vasodilator kinase and forms vasoconstrictor angiotensin II (AngII). AngII has physiological effects and can cause anorexia and muscle wasting in animal model. Therefore, rats lose 18-26% of their body weight during one week by AngII infusion (Brink et al., 1996). In this regard, findings of our study indicated that a weight change in captopril group was less than the placebo group after the course of study. In addition, the patients’ weight increased a little after captopril consumption. 

In the present study we investigated the effects captopril as an Angiotensin-Converting Enzyme Inhibitor on the QoL of patients with cancer cachexia. Our results showed that captopril does not have a significant positive effect on most items of QoL of patients with cancer cachexia. However, fatigue score, physical functioning and global health status significantly improved after 2 months. 

Due to cancer cachexia and end of life stage and also borderline blood pressure of patients, we cannot increase captopril dosage or ACEI in these patients; hence, the lack of effect on QoL might be due to the low concentration of captopril given to patients, which is a limitation in this study. 

Overall there were a number of limiting factors that reduced the impact of the results, of which small number of investigated patients can be mentioned. In addition, relatively short duration of follow ups prevented us from further comparison. Regarding methodology, it should be mentioned that cachexia has multidimensional pathogenesis and we did not mention tumor pathology and self-life style of patients that might have affected the severity of cachexia and quality of life. 

However, the effect of chemotherapy as palliation and for control of cancer and disease related symptoms should be considered. Chemotherapy can control the progression of disease and therefore decrease the release of cytokines which is important in cancer cachexia by increasing the QoL. 

In Conclusion, with respect to the alterations in the patients’ QoL with cancer cachexia after receiving ACE, the authors concluded that there was a clinically-relevant improvement only in the physical functioning, global health status and in the symptom of fatigue parameters. Moreover, the patients’ weight improved after receiving ACEI. Finally, the findings showed that Angiotensin-Converting Enzyme Inhibitor has no significant effect on most items in QoL in cancer cachectic patients. Further studies with larger sample size must be undertaken to assess the results obtained in this study.

## References

[B1] Adigun AQ, Ajayi AA (2001). The effects of enalapril-digoxin-diuretic combination therapy on nutritional and anthropometric indices in chronic congestive heart failure: preliminary findings in cardiac cachexia. Eur J Heart Fail.

[B2] Bosaeus I (2008). Nutritional support in multimodal therapy for cancer cachexia. Support Care Cancer.

[B3] Brink M, Russ Price S, Jacqueline C (2001). Angiotensin II induces skeletal muscle wasting through enhanced protein degradation and down-regulates autocrine insulin-like growth factor I 1. Endocrinology.

[B4] Brink M Jason W, Patrick D (1996). Angiotensin II causes weight loss and decreases circulating insulin-like growth factor I in rats through a pressor-independent mechanism. J Clin Invest.

[B5] Fearon K, Jann A, Vickie B (2013). Understanding the mechanisms and treatment options in cancer cachexia. Nat Rev Clin Oncol.

[B6] Fearon K, Florian S, Stefan DA (2011). Definition and classification of cancer cachexia: an international consensus. Lancet Oncol.

[B7] Inui A (1999). Cancer anorexia-cachexia syndrome. Cancer Res.

[B8] Inui A (2002). Cancer anorexia-cachexia syndrome: current issues in research and management. CA Cancer J Clin.

[B9] Lindberg H, Dorte N, Benny VJ, Jens E, Torben S (2003). Angiotensin converting enzyme inhibitors for cancer treatment?. Acta Oncol.

[B10] Lundholm K, Ulla K, Lena G (2007). Insulin treatment in cancer cachexia: effects on survival, metabolism, and physical functioning. Clin Cancer Res.

[B11] Madeddu C, Mariele D, Filomena P (2012). Randomized phase III clinical trial of a combined treatment with carnitine+ celecoxib±megestrol acetate for patients with cancer-related anorexia/cachexia syndrome. Clin Nutr.

[B12] Montazeri A, Harirchi I, Vahdani M (1999). The European Organization for Research and Treatment of Cancer Quality of Life Questionnaire (EORTC QLQ-C30): translation and validation study of the Iranian version. Support Care Cancer.

[B13] Onder G, Brenda WJHP (2002). Relation between use of angiotensin-converting enzyme inhibitors and muscle strength and physical function in older women: an observational study. Lancet.

[B14] Pallis AG, Mouzas IA (2004). Instruments for quality of life assessment in patients with gastrointestinal cancer. Anticancer Res.

[B15] Rogers ES, Roderick D, MacLeod JS (2011). A randomised feasibility study of EPA and Cox-2 inhibitor (Celebrex) versus EPA, Cox-2 inhibitor (Celebrex), Resistance Training followed by ingestion of essential amino acids high in leucine in NSCLC cachectic patients-ACCeRT Study’. BMC Cancer.

[B16] Sanders PM, Russell ST, Tisdale MJ (2005). Angiotensin II directly induces muscle protein catabolism through the ubiquitin–proteasome proteolytic pathway and may play a role in cancer cachexia. Br J Cancer.

[B17] Small W, Jennifer LJ, Timothy DM (2018). Utility of the ACE inhibitor captopril in mitigating radiation-associated pulmonary toxicity in lung cancer. Am J Clin Oncol.

[B18] Suzuki H, Akihiro A, Haruka A (2013). Cancer cachexia pathophysiology and translational aspect of herbal medicine. Jpn J Clin Oncol.

